# New Appliances and Things Medical

**Published:** 1910-11-26

**Authors:** 


### NEW APPLIANCES & THINGS MEDICAL.

**[We shall be glad to receive at our Office, 28 & 29 Southampton Street, Strand, London, W.C., from the manufacturers, specimens of all new preparations and appliances.]**

### THROAT PASTILLES.

(ALLEN AND HANBURY, LTD., 37 Lombard Street, E.C.)

WE have received from the above firm two samples of the new additions to the various medicated throat pastilles which are already so well known to practitioners. The list of these preparations now includes seventy-seven varieties. The two new additions are (75) formaldehyde and menthol and (77) formaldehyde and ol. cinnamon. Both contain 1 minim of formaldehyde in each pastille; the former, in addition, contains $\frac{1}{40}$ grain of menthol, and the latter $\frac{1}{2}$ minim of cinnamon oil. The dose in each case is from four to six pastilles a day. We have tried both the new varieties, and find them very useful adjuncts to treatment; the pastilles containing the oil of cinnamon are, however, very unpleasant to the taste, and we much doubt if the combination is advantageous, since the disinfective power of cinnamon oil vapour is decidedly low. The menthol pastille, on the other hand, is as palatable as it is possible to make such a mixture, and is likely to be of great service in laryngeal irritation and in cases of hoarseness. The basis of the pastilles is, of course, the special jujube used by the firm in all its other lozenges; it dissolves evenly and smoothly, and forms a tempting-looking sweet which is readily accepted by children.

### STRATHCLYDE FLOOR POLISH.

(THE STRATHCLYDE PAINT COMPANY, Kinning Park, Glasgow.)

THIS floor polish is in every way satisfactory. Its use secured a clean, bright, and dry polish on an ordinary deal floor to which it was applied in the customary way with a flannel mop-cloth. The stretch of floor so covered retained its polish for a long time, and was moderately free from dust, though at first the polish appeared to have a tendency to attract dust, probably owing to a mistake in its application, as later trials showed that it was no more dust-holding than any other floor polish. The charm of this composition is its simplicity. It is ready mixed and merely needs application and some elbow work to produce very satisfactory results. It is free from grease and does not make a slippery floor-covering as so many patent polishes do. The makers claim that it is also a disinfectant. We have had no opportunity of testing its germicidal action as yet, but do not consider that this consideration is likely to have much weight with institutional authorities. They want a good floor polish, not a second-rate floor disinfectant; and from what we have seen of the Strathclyde mixture we have no hesitation in saying that it is a good floor polish. Unfortunately no details as to price are supplied with the sample, and we have therefore no means of comparing it, so far as economy is concerned, with its various rivals on the market.

